# Fold classification based on secondary structure – how much is gained by including loop topology?

**DOI:** 10.1186/1472-6807-6-3

**Published:** 2006-03-08

**Authors:** Jieun Jeong, Piotr Berman, Teresa Przytycka

**Affiliations:** 1 Department of Computer Science and Engineering, The Pennsylvania State University, University Park, USA; 2 National Center for Biotechnology Information, National Library of Medicine, National Institute of Health, Bethesda, USA

## Abstract

**Background:**

It has been proposed that secondary structure information can be used to classify (to some extend) protein folds. Since this method utilizes very limited information about the protein structure, it is not surprising that it has a higher error rate than the approaches that use full 3D fold description. On the other hand, the comparing of 3D protein structures is computing intensive. This raises the question to what extend the error rate can be decreased with each new source of information, especially if the new information can still be used with simple alignment algorithms.

We consider the question whether the information about closed loops can improve the accuracy of this approach. While the answer appears to be obvious, we had to overcome two challenges. First, how to code and to compare topological information in such a way that local alignment of strings will properly identify similar structures. Second, how to properly measure the effect of new information in a large data sample.

We investigate alternative ways of computing and presenting this information.

**Results:**

We used the set of beta proteins with at most 30% pairwise identity to test the approach; local alignment scores were used to build a tree of clusters which was evaluated using a new log-odd cluster scoring function. In particular, we derive a closed formula for the probability of obtaining a given score by chance.Parameters of local alignment function were optimized using a genetic algorithm.

Of 81 folds that had more than one representative in our data set, log-odds scores registered significantly better clustering in 27 cases and significantly worse in 6 cases, and small differences in the remaining cases. Various notions of the significant change or average change were considered and tried, and the results were all pointing in the same direction.

**Conclusion:**

We found that, on average, properly presented information about the loop topology improves noticeably the accuracy of the method but the benefits vary between fold families as measured by log-odds cluster score.

## Background

The problem of structure comparison and protein fold classification is important but also computationally challenging. The structure comparison and structure alignment is inherently more difficult than sequence alignment. In last years, significant progress has been made towards designing algorithms to carry out this task and currently a number of fold comparison methods are known [1-11] and several reviews on these methods have appeared [12-14]. Fold comparison methods can be roughly divided into two groups: slow methods that attempt to compute 3-dimensional alignment with atomic precision and fast screening methods that quickly assess fold similarity without attempting precise alignment. Increasingly hybrid algorithms are applied which use a fast but not accurate method as a preprocessing step which is subsequently followed by a slower but more accurate algorithm that is applied only to the structures selected in the first step. Such two-phase methods become more important as the number of protein structures deposited in PDB [15] approaches 3 × 10^4 ^and steadily increases.

There are two basic approaches that are used in the fast structure similarity scoring methods: indexing/hashing methods [7,8,16,17] and linearization/dynamic programming methods [11,18-21]. These two approaches are quite different in nature. Typically indexing/hashing methods are looking for spatial features of the protein structures that can be easily extracted and compared. Similarity between two structures can be then measured in a number of ways, for example by counting the number of common features. In contrast, linearization methods represent 3D structure as a sequence of segments (for example secondary structures) listed in the order of their appearance in the polypeptide chain. Such linear sequences can be aligned using dynamic programming. An obvious shortcoming of this approach is that there is no guarantee that such alignment is consistent with a structural alignment. However, a number of studies indicate that even secondary structure information alone provides a valuable similarity scoring function [18,19,22,23]. Additional attraction of this method is that while it is likely to produce false positive (proteins with similar secondary structure composition that have significant differences in 3D structure) it is rather unlikely to give false negative (proteins with the same 3D structure that have significantly different secondary structure composition). This makes it a good candidate for a screening method in a hybrid approach discussed above. Another advantage of the linearization method is its applicability to alignment of predicted structural segments [24]. Currently there is a number of algorithms that predict secondary structure segments and the accuracy of these algorithms is quite high [25].

Although these algorithms cannot predict orientations of such secondary structure segments in space, several research groups have begun addressing prediction of supersecondary structures [26-29]. Of important supersecondary structures, one that has attracted most attention is a hairpin which is ubiquitous among the beta folds. It would be expected that adding information about hairpin positionwould significantly increase the power of linear methods at least for *β*-fold class.

With this motivation in mind we extend the linear structure similarity method based on secondary structure [18] by indicating which loops form parts of hairpins (these are short loops that connect the two strands of an anti-parallel *β*-sheet, we refer to them as *closed loops*).

Given a protein structure we represent it as a sequence of letters from alphabet {*E*, *H*, *L*} denoting respectively strand, helix, and loop. Each residue has assigned one letter according to the secondary structure in which it is located and thus the length of the sequence is equal to the length of the protein. Additionally, we add "annotations" that indicate the length and the position of closed loops. We use the term *secondary structure sequence *to refer to those annotated sequences.

We developed a new algorithm for secondary structure recognition based on graph theoretical representation of protein structure.

The annotated secondary structure sequences are then compared by computing maximum score local alignments and subsequently clustered by structural similarity. However, rather than using a specificclustering method, we constructed a tree using weighted pair group method and used tree cluster evaluation method based on [18]. We complement the scoring method proposed in [18] (and used byothers, *e.g*., [30]) by providing a closed mathematical formula for statistical relevance of the scores andprovide a rigorous log-odds score.

The alignment parameters are optimized using a genetic algorithm. On average, we observe a noticeable improvement over the method that does not distinguish between loop types, but the benefits vary between fold families. This suggests that fold or family specific approaches should be more accurate than one size-fit-all alignment method.

## Results

Our results are summarized in Tables 2 and 1. The test set consists of 1183 non-redundant (at most 30% identity) beta proteins where each protein was identified by fold number as assigned by SCOP (see Methods). The test protein belonged to 123 different folds. The pairwise similarity has been computed based on secondary structure and loop information using two scoring functions: CL and NCL. CL scores are computed taking into account loop annotation while NCL scores without them. The precise description of the scoring function designed to obtain an accurate alignment is provided in the Methods section.

Table 2 summarizes the improvement obtained by including loop topology information. This table shows also the contribution of the individual folds to the overall averages. To evaluate this improvement we introduce *average log-odds cluster score*. This way the scoring method introduced in [18] is complemented with a measure of score significance. The complete mathematical derivation of the formula used to obtain the score significance is provided in Additional file 1.

In Table 1, we compare the impact of the secondary structure definition on the results produced by CL and NCL. Here we additionally use our CL and NCL alignments methods in conjunction with the DSSP secondary structure annotation [31]. The results obtained are consistent. Adding loop annotation in each case leads to the same level of improvement. In both tables we also include results from a related algorithm, SSEA [32,33]. In this algorithm, DSSP secondary structure definition is used and no loop topology information is included. Thus it is expected to have a performance closely related NCL results. This indeed is observed, although each method fails for some folds.

**Figure 1:**
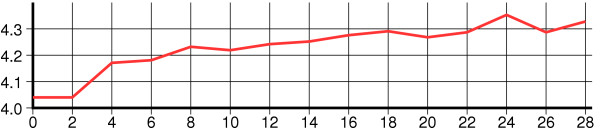
Sums of average unweighted log-odds scores with weighted log-odds scores for different values of *L*. The value for *L *= 0 corresponds to NCL.

The scores obtained with the information about closed loops depend on the limit on the allowed size of closed loops (longer loops are somewhat artificially regarded as open). As demonstrated in Fig. 1, while the best length threshold was 24, we got a marked improvement already for the threshold as low as 4.

## Discussion

We used a large non-redundant set of proteins to create a difficult case for the clustering of folds. While folds represented in the test data by one protein only must have the maximum clustering score, we kept them because they make it more difficult to group other folds in the separate clusters. We used the set of beta proteins because the information about loops in beta hairpins is most relevant for these proteins. The improvement in the clustering accuracy upon adding loop information is independent on the secondary structure recognition algorithm used.

It was not obvious how to score the additional loop information. Incorrect scoring may actually worsen the alignment relative to what can be obtained without the loop annotation. Therefore, we used a hybrid, piecewise linear formula, that that gives "full credit" to closed loop up to a certain length threshold and gradually decreases the score for longer loops. Then a genetic algorithm was used to select parameters for this alignment algorithm. Usually in such a case, there is a concern of overfitting. There are several reasons for which this is not a potential problem in our parameters adjustment. First, we have a very small number of parameters relatively to the number of proteins and number of folds. Second, we used only about half of the proteins in the set for the training purpose. Finally, for the fairness of the comparison between CL and NCL we optimized also the parameters for the NCL version of the program. This allowed us also to compare the results of so optimized NCL alignment with the alternative alignment method implemented in the program SSEA [32,33]. SSEA uses DSSP to recognize secondary structures and has no information about loop topology. The results of SSEA, our alignment program with DSSP structure determination and our alignment program with our own structure determinations were almost identical – on the average. We also cross-validated the results using randomly selected protein sets.

## Conclusion

We studied the question how much secondary structure based fold recognition can be improved by adding the information about the loop topology. Here by the loop topology we understood simple classification of loops between closed loops (loops of length at most *L *which connect two antiparallel strands) and open loops (a broad class containing all other loops). The information about loop length was also included. We observed noticeable improvement over an algorithm that only uses secondary structure types and lengths for *L *as small as four. In practice, this corresponds to including hairpin information. The full improvement is obtained when we take into account only the loops of length up to 24, which means that only the loops of length up to 12 get "full credit". It appears that the improvement was dominated by hairpins, but considering loops of larger length does not decrease the improvement.

The improvements resulting from including loop topology information did not distribute uniformly among protein folds. Indeed, large improvements were experienced by ca. 20% of the folds, while ca. 3% of them got worse. Furthermore, different families responded best to a different set of parameters (e.g. different values of *L*). This suggests that fold specific approach is more accurate than one-size-fit all approach. To perform the study, we developed a number of new algorithms including a new graph theory based secondary structure recognition algorithm, genetic algorithm for parameter optimization and, most importantly, complemented existing cluster evaluation method with more rigorous scoring.

In a future work, we plan to extend this approach to add other supersecondary structure elements like beta-alpha-beta motif, Greek key motif etc.

## Methods

Our general procedure is as follows. We first collect the set of proteins from PDB that were identified in ASTRAL file for the beta class that has at most 30% aligned pairwise identity [34]. Initially only a set of 631 proteins of length between 70 to 140 was used for the training purpose. Once parameters have been adjusted, we performed the clustering on the full file.

For each protein, our secondary structure recognition program read the coordinates of the atoms on the protein backbone and produces the file of secondary structure sequences. In the recognition of the secondary structure segments, we tried to be as close to standard textbook descriptions as possible, and thus our primary criterion was the topology of the hydrogen bonds between backbone atoms (see subsection Secondary structure identification). For comparison, we also performed the experiment with DSSP secondary structure annotation.

A closed loop is a loop that starts and ends at one of the ends of an anti-parallel beta sheet. This intuitive definition has to be relaxed, because part of that loop can be included in a strand of the adjacent sheet, so a "closed loop" may include residues that are not classified as *L*. We defined closed loops in terms of the "innermost" hydrogen bonds of anti-parallel *β*-sheets; such loops may contain residues that participate in other secondary structures, but not other closed loops. For example, if the innermost hydrogen bond is between residues 52 and 70, we alter the symbol at position (52 + 70)/2 = 61 by giving it subscript 70 - 52 = 18.

Next, (see subsection Alignment scores) given a file of secondary structure sequences, we compute the pairwise similarity score and produces the matrix of alignment scores. Alignment score is defined with a number of parameters. To separate the improvement that comes from the choice of parameters and an improvement that comes from the choice of the method (CL versus NCL) we separately optimized these parameters. In other words, the most fair comparison between CL and NCL requires that each is at its best.

Next, (see subsection Clustering and cluster scoring) we build the tree of clusters using the weighted pair group method and measure the quality of the prediction by giving the comparison scores between a cluster in our tree and the fold class in SCOP. SCOP is the structural classification of proteins of all known protein structures. It categorizes protein domains at the level of class, fold, superfamily and family based on homologous sequences, three dimensional structure, information about evolution, and human judgment.

Here we consider only fold class for our benchmarking because it is exclusively based on three dimensional structure. We compute comparison scores of fold sets and the scores of random sets of the same size, and thus we obtain the log-odds score. Finally, we compute the raw score and log-odds score for each fold in the data set.

This process was repeated by our genetic algorithm and the average log-odds score was used as the feedback information when different parameters vectors were compared. We also used more exhaustive search near among vectors that were close to the best ones found by the genetic algorithm. We have altogether 9 parameters, and to avoid overfitting, we restricted their values to small sets (*e.g*., integers from the list 4, 8, 12, ..., 28). In the training process only a subset of proteins (about 50%) and protein folds was used.

### Secondary structure identification

We developed a method of secondary structure classification and automatic recognition of closed loops. We also performed the same experiment using the DSSP [31] secondary structure assignment where using the loop annotation transferred from our recognition algorithm. While there is no benefit in replacing the DSSP approach with our algorithm for secondary structure recognition alone, it opens the door for modifications that allow capturing other structural motifs. Recognition of closed loops is the first step in this direction. Our method of secondary structure identification can be described as follows. We first compute the hydrogen bonds between atoms on the protein backbone. For each hydrogen bond, we store *bond pair *(*a*, *b*), where *a *and *b *are the numbers of residues it is connecting; we will always have *a *<*b*. Next, we define a graph in which vertices are the bond pairs and the edges form the set

{[(*a*, *b*), (*c*, *d*)] : |*a *- *c*| ≤ 2 and |*b *- *d*| ≤ 2}.

Then, the alpha helices and beta sheets are identified as certain connected components of this graph. Components representing a particular kind of the secondary structures are identified using an appropriate rule. The rule of an alpha helix is that for each bond pair (*a*, *b*) we have *b *- *a *= 4. If the bond satisfies this rule, we give 1, if not, we give -1. A connected component passes the alpha-helix test if the total score is at least 0 (the majority rule).

**Figure 2:**
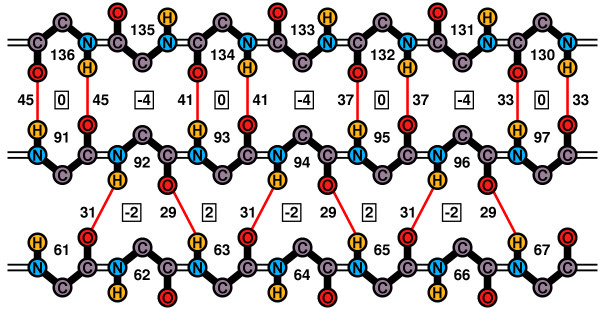
Ideal cases of a parallel and anti-parallel beta sheets. Residue numbers are surrounded by the backbone atoms of the respective residue, differences of hydrogen bonds are positioned next to the respective bonds and second differences are placed in boxes.

Rules for beta sheets compare bond pairs that are adjacent in the graph we have described. Therefore we start by sorting the bond pairs of a connected component by the lower residue number, tie-breaking with the higher number – adjacent bonds become consecutive in this order. This gives a sequence (*a*_1_, *b*_1_), ..., (*a*_*n*_, *b*_*n*_). We convert it to a sequence of differences, *d*_1 _..., *d*_*n *_where *d*_*i *_= *b*_*i *_- *a*_*i*_. Subsequently we convert it to the second sequence of differences *e*_1_, ..., *e*_*n*-1 _where *e*_*i *_= *e*_*i*+1 _- *e*_*i*_. As illustrated in Fig. 2 the second sequence of differences of a perfect anti-parallel beta sheet is 0, -4, 0, -4, ... or -4, 0, -4, 0, ... and for a perfect parallel beta sheet it is 2, -2, 2, -2, ... or -2, 2, -2, 2, .... When a term of the second sequence follows the rule (*e.g*., 0 after -4 or -4 after 0 in an anti-parallel sheet) we score 1, if it does not follow the rule but belongs to allowed range (*e.g*., {0, -4} for an anti-parallel sheet) we score 0, otherwise we score -1. Again, the condition to pass the test is to score at least 0.

One concern with the above definition may be whether it is possible to accumulate lots of violations and still have positive score or whether it is possible that negative scores accumulate on one or both endpoints of the component so that the sum is negative while the components contained a perfectly correct secondary structure as a subcomponent. In practice, it is hardly possible to combine many "violations" with positive scores. The only major exception we have encountered is a bacteriophage parallel beta helix, in which hydrogen bonds of all the strands in the beta helix, as well as the hydrogen bonds of the beta turns coalesced into one large connected components. Because all structures from that fold were affected very similarly, this anomaly was not detrimental in our application.

The second apparent anomaly of this method would occur for the tightest possible hairpin loops: the innermost hydrogen bond connects adjacent residues; these residues should be excluded from the adjacent anti-parallel beta sheet because they fail the dihedral angle condition. As a result, this definition would not inform NL method about that loop. This anomaly was eliminated by applying the dihedral angle test at the ends of computed strands; when the test failed, the pairs of the bonds adjacent to such a residue were removed from the respective component and the test was applied again.

### Alignment scores

To define the alignment score, we assign scores to substitution and gaps. Below we define the parameters that we were using, and the selected values of those parameters are given at the end of this subsection.

We used a fixed positive score *E *for an equal substitution, a negative difference cost *D *for *L*/*H *and *L*/*E *substitutions and a prohibitively low score of *E*/*H *replacement.

The score of a gap of length ℓ is min(ℓ*L*_*s*_, *L*_*o *_+ ℓ*L*_*e*_), where *L*_*s *_is the price of extending a short gap, *L*_*o *_is the price of *opening *a long gap, and *L*_*e *_is the price of *extending *a long gap. This *piecewise linear *gap penalty is easy to compute.

We have chosen this hybrid formula because we expected that  the lengths of loops and the secondary structures have some small variability when we compare homologous structures from the same fold, and  some loops may be replaced with quite a long sequence that contains one or more secondary structures.

Short gap pricing is appropriate for  and long gap pricing (high opening cost, small extension cost) is appropriate for .

The highest scoring local alignment for two secondary structure sequences can be efficiently computed using the dynamic programming method that is essentially the same as the one used by Gotoh ([35]). In particular, given two sequences (*a*_1_, ..., *a*_*m*_) and (*b*_1_, ..., *b*_*n*_) we define *subproblems *of the form *S*(*i*, *j*, *s*) where 0 ≤ *i *≤ *m*, 0 ≤ *j *≤ *n *and 0 ≤ *s *≤ 1; *S*(*i*, *j*, *s*) is the highest local alignment score of sequences (*a*_1_, ..., *a*_*i*_) and (*b*_1_, ..., *b*_*j*_), either without any restriction (state *s *= 0) or under the assumption that the alignment ends with a block of gaps that are priced according to the long gap penalty method (state *s *= 1). We can define *S *recursively: if *i *< 0 or *j *< 0 we have *S*(*i*, *j*, *s*) = -∞ and

S(i,j,0)=max⁡{0S(i,j,1)S(i,j−1,0)+SS(i−1,j,0)+SS(i−1,j−1,0)+Subst(ai,bj)S(i,j,1)=max⁡{S(i,j,0)+LoS(i,j−1,1)+LeS(i−1,j,1)+Le
 MathType@MTEF@5@5@+=feaafiart1ev1aaatCvAUfKttLearuWrP9MDH5MBPbIqV92AaeXatLxBI9gBaebbnrfifHhDYfgasaacH8akY=wiFfYdH8Gipec8Eeeu0xXdbba9frFj0=OqFfea0dXdd9vqai=hGuQ8kuc9pgc9s8qqaq=dirpe0xb9q8qiLsFr0=vr0=vr0dc8meaabaqaciaacaGaaeqabaqabeGadaaakeaafaqabeqacaaabaGaem4uamLaeiikaGIaemyAaKMaeiilaWIaemOAaOMaeiilaWIaeGimaaJaeiykaKIaeyypa0JagiyBa0MaeiyyaeMaeiiEaG3aaiqaaeaafaqaaeqbbaaaaeaacqaIWaamaeaacqWGtbWucqGGOaakcqWGPbqAcqGGSaalcqWGQbGAcqGGSaalcqaIXaqmcqGGPaqkaeaacqWGtbWucqGGOaakcqWGPbqAcqGGSaalcqWGQbGAcqGHsislcqaIXaqmcqGGSaalcqaIWaamcqGGPaqkcqGHRaWkcqWGtbWuaeaacqWGtbWucqGGOaakcqWGPbqAcqGHsislcqaIXaqmcqGGSaalcqWGQbGAcqGGSaalcqaIWaamcqGGPaqkcqGHRaWkcqWGtbWuaeaacqWGtbWucqGGOaakcqWGPbqAcqGHsislcqaIXaqmcqGGSaalcqWGQbGAcqGHsislcqaIXaqmcqGGSaalcqaIWaamcqGGPaqkcqGHRaWkcqWGtbWucqWG1bqDcqWGIbGycqWGZbWCcqWG0baDcqGGOaakcqWGHbqydaWgaaWcbaGaemyAaKgabeaakiabcYcaSiabdkgaInaaBaaaleaacqWGQbGAaeqaaOGaeiykaKcaaaGaay5EaaaabaGaem4uamLaeiikaGIaemyAaKMaeiilaWIaemOAaOMaeiilaWIaeGymaeJaeiykaKIaeyypa0JagiyBa0MaeiyyaeMaeiiEaG3aaiqaaeaafaqaaeWabaaabaGaem4uamLaeiikaGIaemyAaKMaeiilaWIaemOAaOMaeiilaWIaeGimaaJaeiykaKIaey4kaSIaemitaW0aaSbaaSqaaiabd+gaVbqabaaakeaacqWGtbWucqGGOaakcqWGPbqAcqGGSaalcqWGQbGAcqGHsislcqaIXaqmcqGGSaalcqaIXaqmcqGGPaqkcqGHRaWkcqWGmbatdaWgaaWcbaGaemyzaugabeaaaOqaaiabdofatjabcIcaOiabdMgaPjabgkHiTiabigdaXiabcYcaSiabdQgaQjabcYcaSiabigdaXiabcMcaPiabgUcaRiabdYeamnaaBaaaleaacqWGLbqzaeqaaaaaaOGaay5Eaaaaaaaa@AEE1@

We conveyed the information about the closed loops as follows. First, we defined intervals of hydrogen bonds: a bond between residue *i *and residue *j *defines integer interval {*i *+ 1, *i *+ 2, ..., *j *- 1}. We considered the family of intervals of the hydrogen bonds from anti-parallel *β*-sheets, and we removed those that overlap shorter intervals from that family and those that exceed a length threshold. The remaining intervals were assumed to be closed loops.

A closed loop with interval {*i *+ 1, ..., *j *- 1} is represented as an annotation *j *- *i *at position ⌊(*i *+ *j*)/2⌋. Almost always that position is indeed in a loop, so instead of symbol *L *we have symbol *L*^*j*-*i*^; sometimes it is *H*^*j*-*i *^– a single turn of an *α*-helix may be a part of a hairpin loop.

A homologous pair of closed loops is flanked by pairs of *β*-strands that are also homologous to each other,so we expected the annotations of these loops to align.

Therefore we can alter the formula for scoring of substitutions to score the alignments of closed loops, as aresult we do not have to change the dynamic programming that calculates the local alignment score. The only modification is hidden in the definition of *Subst*(*a*, *b*).

More precisely, if *a *is an annotated symbol *X*^*k*^, we define *s*(*a*) = *X *and ℓ(*a*) = *k*, if *a *is not an annotated symbol, we have ℓ(*a*) = ⊥ (undefined). If ℓ(*a*) = ⊥ or ℓ(*b*) = ⊥ then *Subst*(*a*, *b*) = *Subst*(*s*(*a*), *s*(*b*)).

Otherwise,

*Subst*(*a*, *b*) = *Subst*(*s*(*a*), *s*(*b*)) + *Premium*(ℓ(*a*), ℓ(*b*)).

In turn, *Premium*(*i*, *j*) is defined with three parameters: *M*, the maximum premium, *P*, the penalty for difference in length and *L*, the largest length of a loop that may get a premium.

We decided to decrease the premium for alignment of closed loops with length penalty defined as

*LengthPen*(*i*, *j*) = max(*i *+ *j *- *L*, 0) × *M*/*L*.

Homologous loops should have similar lengths, so we used a penalty for the difference in lengths, |*i *- *j*| × *P*. The overall formula for the premium is

*Premium*(*i*, *j*) = max{0, *M *- *LengthPen*(*i*, *j*) - |*i *- *j*|*D*}.

Finally, we had to adjust the scores for the length of the compared proteins. Note that a short protein may find a highly similar fragment in a long protein purely by coincidence, especially that our sequences have low information content: only 3 symbols that have long series of repetitions. Consequently, we should decrease the weight for such a score. Suppose that the score in question is *s*, the length of the shorter protein is ℓ and the length of the longer protein is *L*. Obvious methods of computing the adjusted score, like *s*/*L*, *s*/ℓ and *s*/ℓL
 MathType@MTEF@5@5@+=feaafiart1ev1aaatCvAUfKttLearuWrP9MDH5MBPbIqV92AaeXatLxBI9gBaebbnrfifHhDYfgasaacH8akY=wiFfYdH8Gipec8Eeeu0xXdbba9frFj0=OqFfea0dXdd9vqai=hGuQ8kuc9pgc9s8qqaq=dirpe0xb9q8qiLsFr0=vr0=vr0dc8meaabaqaciaacaGaaeqabaqabeGadaaakeaadaGcaaqaaiabloriSjabdYeambWcbeaaaaa@2F19@ yielded inferior results, so we decided to use formula *s*ℓ^-*g*^*L*^-*h *^where *g *and *h *were additional parameters of the similarity function.

To find optimum values for the parameters, we rather arbitrarily fixed *E *to be 16, and then we search for the best values of *D*, *S*, *L*_*o *_and *L*_*e*_, and in the case when loop information is considered, the values of *M*, *L *and *P*.

For each of the parameters we gave 3 widely dispersed values and this created an initial population of 3^4 ^= 27 (or 3^7 ^= 243) parameter vectors. For each vector we computed the resulting average log-odds score – on the sample of 631 medium length domains a single computation was taking about a minute. Next we designed a genetic algorithm, in which we were selecting randomly two vectors, with the bias for the top scoring vectors, and computed a random linear combination for each of the parameters, with the bias for the arithmetic average. This process increased the value of the best average log-odds score. We finish by taking the best vector and trying 3–4 closely spaced values for each of the parameters.

To avoid over-fitting (and save time) we restricted the values as follows: *E *= 16, *D *and *S *were multiples of 5, *L*_*o *_as a multiple of 10, *L*_*e *_was a multiple of 2, *a*, *b *were multiples of 1/5.

Our best parameter vector for the case without loop annotations was

(*E*, *D*, *S*, *L*_*o*_, *L*_*e*_, *g*, *h*) = (16, -35, -25, -170, -2, 0, 0.6),

and in the case with the annotation it was

(*E*, *D*, *S*, *L*_*o*_, *L*_*e*_, *M*, *P*, *L*, *g*, *h*) = (16, -35, -25, -170, -2, 120, 16, 24, 0.0, 0.8).

### Clustering and cluster-scoring

We used the weighted pair group method for clustering which is applied to the matrix of alignment scores (see for example [36]). In this method we start with 1-element clusters and then we keep merging a pair of clusters *A*, *B *with the maximum average of "similarity score of *a *from *A *and *b *from *B*" (where score is given by a method that we are testing). This rule defines a rooted binary tree where each internal node defines a cluster.

Given this tree we can calculate first the raw score of a set *S *(*e.g*., proteins from some fold).

Following [18], the raw score of a set *S*, *σ*(*S*) in the tree is computed as follows. For each node *v *of the tree *T *define the weight of *S *in the cluster *C*, *w*_*s*_(*C*) as follows: if *C *is a subset of *S*, *w*_*s*_(*C*) = 1, if *C *is disjoint with *S*, *w*_*s*_(*C*) = 0 and in other cases *C *is formed as a union of smaller clusters, *C*_0 _and *C*_1 _and its weight is the average (*w*_*s*_(*C*_0_) + *w*_*s*_(*C*_1_))/2. Then, for a pair of elements of *S *we define the weight of this pair in the cluster as the weight of least common ancestor of the two elements of the pair. Finally, the score of set *S *is the average of the weights of all pairs of elements from *S*.

If the raw score of a set *S *is high (close to one) then it indicates that *S *forms in the tree *T *in a good cluster independently on the shape of the tree *T*. However if the score of *S *close to 0.5 or less, then the fact whether or not such a score indicates a reasonable clustering depends on the topology of the tree. For example, consider two trees with 1024 nodes. In the first one the average distance of leaves to the root is maximal, *i.e*. it equals ca. 512, while in the second one it is minimal, *i.e*. it equals 10. One can show that the expected score of a random set of 3 nodes is 0.502 in the first tree and 0.011 in the second tree.

Therefore we use a log-odd type scoring function where the clustering score of a set *S *in a tree *T *is compared to a score of a random set of the same size and in the same tree. In Additional file 1 we prove the following theorem:

**Theorem: **If *F *is a random set of leaves with *k *elements, then the expected score of *F *on a tree *T *is equal to:

S(T,k)=E[σ(F)]=k−2n−2+n−k(n−1)(n−2)α(T)
 MathType@MTEF@5@5@+=feaafiart1ev1aaatCvAUfKttLearuWrP9MDH5MBPbIqV92AaeXatLxBI9gBaebbnrfifHhDYfgasaacH8akY=wiFfYdH8Gipec8Eeeu0xXdbba9frFj0=OqFfea0dXdd9vqai=hGuQ8kuc9pgc9s8qqaq=dirpe0xb9q8qiLsFr0=vr0=vr0dc8meaabaqaciaacaGaaeqabaqabeGadaaakeaacqWGtbWucqGGOaakcqWGubavcqGGSaalcqWGRbWAcqGGPaqkcqGH9aqpieqacqWFfbqrcqGGBbWwiiGacqGFdpWCcqGGOaakcqWGgbGrcqGGPaqkcqGGDbqxcqGH9aqpdaWcaaqaaiabdUgaRjabgkHiTiabikdaYaqaaiabd6gaUjabgkHiTiabikdaYaaacqGHRaWkdaWcaaqaaiabd6gaUjabgkHiTiabdUgaRbqaaiabcIcaOiabd6gaUjabgkHiTiabigdaXiabcMcaPiabcIcaOiabd6gaUjabgkHiTiabikdaYiabcMcaPaaacqGFXoqycqGGOaakcqWGubavcqGGPaqkaaa@56CD@

where *n *= |ℒ
 MathType@MTEF@5@5@+=feaafiart1ev1aaatCvAUfKttLearuWrP9MDH5MBPbIqV92AaeXatLxBI9gBamrtHrhAL1wy0L2yHvtyaeHbnfgDOvwBHrxAJfwnaebbnrfifHhDYfgasaacH8akY=wiFfYdH8Gipec8Eeeu0xXdbba9frFj0=OqFfea0dXdd9vqai=hGuQ8kuc9pgc9s8qqaq=dirpe0xb9q8qiLsFr0=vr0=vr0dc8meaabaqaciaacaGaaeqabaWaaeGaeaaakeaaimaacqWFsectaaa@376E@(*T*)| is the number of leaves of *T *and *α*(*T*) is the average distance of leaves from the root. For a *k *element set *S *define log-score of a set *S*, as

log⁡−score(S)=log⁡σ(S)S(T,k)
 MathType@MTEF@5@5@+=feaafiart1ev1aaatCvAUfKttLearuWrP9MDH5MBPbIqV92AaeXatLxBI9gBaebbnrfifHhDYfgasaacH8akY=wiFfYdH8Gipec8Eeeu0xXdbba9frFj0=OqFfea0dXdd9vqai=hGuQ8kuc9pgc9s8qqaq=dirpe0xb9q8qiLsFr0=vr0=vr0dc8meaabaqaciaacaGaaeqabaqabeGadaaakeaacyGGSbaBcqGGVbWBcqGGNbWzcqGHsislcqqGZbWCcqqGJbWycqqGVbWBcqqGYbGCcqqGLbqzcqGGOaakcqWGtbWucqGGPaqkcqGH9aqpcyGGSbaBcqGGVbWBcqGGNbWzdaWcaaqaaGGaciab=n8aZjabcIcaOiabdofatjabcMcaPaqaaiabdofatjabcIcaOiabdsfaujabcYcaSiabdUgaRjabcMcaPaaaaaa@4BA3@

where *σ*(*S*) is the raw score of the set *S *as defined above.

## Contributions of authors

JJ conceived and developed the new method of secondary structure identification and the formula for the alignment scores. JJ developed the software that integrated these elements with the computation of cluster scores and designed the genetic algorithm. JJ analyzed data and wrote the initial version of the manuscript. JJ and PB performed the comparison with SSEA and DSSP and cross-validation and created the manuscript figures. JJ and PB worked out the formula for the expected cluster score. TP conceived the problem, provided biological literature, helped to interpret the data, supervised the project and provided the support. The idea of log-odds cluster scoring originated in joint discussions.

## Supplementary Material

Additional File 1 Click here for file

## Figures and Tables

**Table 1:** Clustering scores of various methods.

Sample	Size	averaging method	SSEA	DSSP		Ours	
				
				NCL	CL	NCL	CL
ALL	1183	U	2.30	2.27	2.49	2.36	2.50
		R	2.08	2.07	2.27	2.09	2.26
		L	1.71	1.70	1.84	1.68	1.85
MEDIUM	631	U	1.82	1.87	1.98	1.81	2.04
		R	1.62	1.66	1.77	1.59	1.78
		L	1.18	1.18	1.27	1.11	1.26
LONG	475	U	1.96	2.03	2.05	1.92	2.00
		R	1.81	1.85	1.90	1.76	1.86
		L	1.64	1.68	1.73	1.61	1.71
RANDOM	591	U	1.76	1.77	1.87	1.88	1.98
		R	1.64	1.63	1.73	1.71	1.81
		L	1.42	1.37	1.47	1.43	1.53

Average log-odds score of various clustering functions. Sample MEDIUM consists of those protein domains in ALL that have between 70 and 140 residues, and LONG are those that are longer. RANDOM is the average of 40 samples obtained by splitting ALL in a random fashion into equal parts (on the average). Averaging methods: U is unweighted, R is weighted with the root of fold size and L is weighted with the fold size (in a sample); in each case folds that have fewer than 2 representatives in a sample are excluded. SSEA is the score computed by SSEA program from DSSP output, DSSP is the score obtained from DSSP output and our alignment program, "ours" uses our structure determination and our alignment programs. Our annotations of closed loops were transferred to DSSP output to obtain CL version of that score.

**Table 2:** Raw scores and log-odds scores for individual folds.

fold number	fold size	score			Log-odds score			impact on the average		
					
			ours			ours				
								
		SSEA	NCL	CL	SSEA	NCL	CL	U	R	L
1	242	0.462	0.461	0.493	0.748	0.751	0.813			2
2	27	0.054	0.089	0.077	0.293	0.832	0.659			
3	11	0.096	0.087	0.096	1.276	1.238	1.274			
4	2	1.000	1.000	1.000	3.952	4.030	3.945			
6	40	0.199	0.303	0.257	1.366	1.815	1.619		-1	-1
7	15	0.299	0.378	0.315	2.300	2.583	2.345			
8	3	0.333	0.334	0.334	2.811	2.888	2.804			
11	9	0.112	0.221	0.377	1.494	2.238	2.706	1	1	
12	4	0.505	0.334	0.151	3.186	2.843	1.975	-2	-1	
15	3	0.062	0.378	1.000	1.137	3.010	3.903	2	1	
17	3	0.521	0.833	0.833	3.257	3.802	3.720			
18	29	0.140	0.126	0.169	1.215	1.142	1.399		1	1
19	6	0.152	0.167	0.177	1.907	2.069	2.056			
21	3	0.339	0.333	0.333	2.827	2.886	2.804			
22	7	1.000	0.717	0.719	3.756	3.487	3.420			
23	6	0.304	0.276	0.677	2.601	2.571	3.396	2	1	
24	5	0.183	0.473	0.850	2.131	3.149	3.661	1		
26	5	0.104	0.104	0.150	1.569	1.633	1.929			
29	33	0.361	0.258	0.581	2.084	1.779	2.557	1	3	4
30	17	0.286	0.299	0.456	2.200	2.292	2.663		1	1
31	2	0.035	0.016	0.009	0.604	0.000	0.000			
33	7	0.404	0.303	0.839	2.850	2.627	3.574	2	2	1
34	63	0.340	0.160	0.271	1.582	0.846	1.354	1	3	5
35	3	0.104	0.043	0.021	1.648	0.837	0.063	-1	-1	
36	20	0.638	0.492	0.680	2.927	2.709	2.986		1	
37	2	0.008	0.004	0.000	0.000	0.000	0.000			
38	11	0.610	0.228	0.418	3.129	2.201	2.745	1	1	1
39	2	1.000	1.000	1.000	3.952	4.030	3.945			
40	90	0.244	0.277	0.301	0.973	1.112	1.182			1
41	2	1.000	1.000	1.000	3.952	4.030	3.945			
42	24	0.389	0.309	0.447	2.340	2.149	2.475		1	1
43	27	0.300	0.244	0.246	2.016	1.844	1.813			
44	3	0.833	0.418	0.333	3.727	3.112	2.804			
45	4	0.376	1.000	1.000	2.890	3.941	3.862			
46	2	0.001	0.039	0.000	0.000	0.787	0.000	-1		
47	32	0.753	0.424	0.395	2.838	2.296	2.189			
49	2	1.000	1.000	1.000	3.952	4.030	3.945			
50	11	0.382	0.352	0.331	2.662	2.635	2.513			
51	4	0.199	0.502	0.531	2.254	3.251	3.230			
52	10	0.353	0.256	0.308	2.615	2.351	2.471			
53	3	0.003	0.064	0.500	0.000	1.235	3.210	4	2	1
55	30	0.531	0.492	0.607	2.526	2.483	2.656			
56	2	1.000	1.000	1.000	3.952	4.030	3.945			
57	3	1.000	1.000	1.000	3.910	3.984	3.903			
58	3	0.335	1.000	1.000	2.815	3.984	3.903			
60	22	0.489	0.488	0.478	2.613	2.652	2.587			
61	9	0.127	0.084	0.352	1.623	1.264	2.637	3	3	2
62	2	1.000	1.000	1.000	3.952	4.030	3.945			
63	2	1.000	1.000	1.000	3.952	4.030	3.945			
64	2	0.016	1.000	1.000	0.000	4.030	3.945			
65	2	0.126	0.001	0.063	1.877	0.000	1.174	2	1	
66	5	1.000	0.900	1.000	3.830	3.793	3.824			
67	2	0.002	0.016	0.000	0.000	0.000	0.000			
68	13	0.550	0.382	0.552	2.966	2.653	2.964			
69	23	0.747	0.711	0.750	3.014	3.005	3.015			
70	5	0.475	0.368	0.591	3.086	2.900	3.297			
71	24	0.342	0.148	0.281	2.212	1.412	2.010	1	2	2
72	10	0.602	0.454	0.533	3.148	2.922	3.021			
74	4	1.000	1.000	1.000	3.869	3.941	3.862			
76	2	0.000	0.531	0.000	0.000	3.397	0.000	-8	-3	-1
77	5	0.331	0.259	0.303	2.725	2.549	2.629			
80	18	0.302	0.305	0.431	2.228	2.282	2.582		1	
81	9	0.559	0.643	0.591	3.106	3.304	3.156			
82	35	0.173	0.205	0.399	1.311	1.512	2.143	1	3	3
83	2	0.258	0.031	0.012	2.597	0.565	0.000	-1		
84	14	0.165	0.143	0.225	1.735	1.638	2.038		1	
85	14	0.076	0.070	0.141	0.959	0.924	1.571	1	1	1
86	3	0.355	1.000	1.000	2.874	3.984	3.903			
87	4	0.167	0.208	0.501	2.080	2.373	3.171	1	1	
88	3	0.089	0.011	0.048	1.489	0.000	0.870	2	1	
91	2	0.750	1.000	1.000	3.664	4.030	3.945			
92	2	0.009	0.008	0.062	0.000	0.000	1.172	2	1	
93	2	1.000	1.000	1.000	3.952	4.030	3.945			
104	2	0.094	0.125	0.500	1.585	1.951	3.252	3	1	
106	3	0.006	0.009	0.003	0.000	0.000	0.000			
108	2	0.250	0.002	0.000	2.566	0.000	0.000			
113	4	1.000	0.875	1.000	3.869	3.807	3.862			
118	2	1.000	1.000	1.000	3.952	4.030	3.945			
121	49	0.211	0.246	0.305	1.287	1.465	1.654		1	1
122	5	0.157	0.120	0.188	1.978	1.779	2.153			
125	2	1.000	1.000	1.000	3.952	4.030	3.945			
Unweighted avg.		0.417	0.434	0.501	2.300	2.368	2.500			
size MathType@MTEF@5@5@+=feaafiart1ev1aaatCvAUfKttLearuWrP9MDH5MBPbIqV92AaeXatLxBI9gBaebbnrfifHhDYfgasaacH8akY=wiFfYdH8Gipec8Eeeu0xXdbba9frFj0=OqFfea0dXdd9vqai=hGuQ8kuc9pgc9s8qqaq=dirpe0xb9q8qiLsFr0=vr0=vr0dc8meaabaqaciaacaGaaeqabaqabeGadaaakeaadaGcaaqaaiabdohaZjabdMgaPjabdQha6jabdwgaLbWcbeaaaaa@3261@ weighted avg.		0.389	0.378	0.453	2.080	2.088	2.263			
weighted avg.		0.376	0.351	0.421	1.705	1.675	1.853			

Raw scores and log-odds scores for 81 folds that had more than one representative in our data. SSEA score was obtained by taking structure determinations of DSSP and computing the scores using the publically available binary code of SSEA program. Our scores were computed using our alignment program and using our structure determinations, which were similar but not identical to DSSP. Averages are: unweighted (U), root weighted (R) – fold with k proteins get weight k
 MathType@MTEF@5@5@+=feaafiart1ev1aaatCvAUfKttLearuWrP9MDH5MBPbIqV92AaeXatLxBI9gBaebbnrfifHhDYfgasaacH8akY=wiFfYdH8Gipec8Eeeu0xXdbba9frFj0=OqFfea0dXdd9vqai=hGuQ8kuc9pgc9s8qqaq=dirpe0xb9q8qiLsFr0=vr0=vr0dc8meaabaqaciaacaGaaeqabaqabeGadaaakeaadaGcaaqaaiabdUgaRbWcbeaaaaa@2E26@ and weighted (L), where the weight is k. "Impact on the average" shows how the respective average would change if all other folds had identical scores; we multiply this change by 200 and round toward 0; zeroes are not shown.
