# Supplementary material for: Fold classification based on secondary structure – how much is gained by including loop topology?
Source: BMC Struct Biol. 2006 Mar 8;6:3. doi: 10.1186/1472-6807-6-3 (PMC1434743; doi:10.1186/1472-6807-6-3)
Supplement: Additional File 1 [file 1472-6807-6-3-S1.pdf]

Expected cluster score of a random set

Here we derive a closed formula for the expected score, $S(k,T)$ of a randomly selected cluster of k leaves in a fixed tree T. The expected score depends on the size of the cluster and the shape of the tree.

Given a tree T with root we can define for every node u

$\lambda(u)$	the left child of u
$\rho(u)$	the right child of u
$A(u,v)$	the least common ancestor of u and v
$\mathcal{L}(u)$	the leaves of the subtree of u
$s(u)$	$= \lvert\mathcal{L}(u)\rvert$
$\alpha(T)$	the average distance of leaves of T to the root

For $v \in \mathcal{L}(u)$ we define $\varepsilon(u,v)$ as follows: if u is a leaf then $u = v$ and $\varepsilon(u,v) = 1$; otherwise for a child w of u (w s.t. $w \in \{\lambda(u), \rho(u)\}$) we have $v \in \mathcal{L}(w)$ and we define $\varepsilon(u,v) = \frac{1}{2}\varepsilon(w,v)$.

Observation.

$$\sum_{v\in\mathcal{L}(u)} \varepsilon(u,v) = 1.$$

Let $\mathcal{L} = \mathcal{L}(root(T))$ and $n = s(root(T))$. Want to compute $S(T,k)$, the expected score of a random $F \subset \mathcal{L}$ that has k elements.

To define the score of $F \subset \mathcal{L}$, we first define node weight for the nodes of T: for a leaf u, $\mathbf{w}(u) = [\![u \in F]\!]$ where $[\![\mathit{formula}]\!]$ is 1 if *formula* is true and 0 if it is false; otherwise $\mathbf{w}(u) = \frac{1}{2}(\mathbf{w}(\lambda(u)) + \mathbf{w}(\rho(u)))$. For a pair $\{u,v\} \subset F$, the score $\sigma(u,v)$ is defined as $\mathbf{w}(A(u,v))$; in turn, $\sigma(F)$ is the average of $\sigma(u,v)$ over unordered pairs contained in F.

We can extend the definition of $\sigma(u,v)$ to all pairs in $\mathcal{L}$, $\sigma(u,v)$ is zero if $\{u,v\} \not\subset F$, so it equals $\mathbf{w}(u)\mathbf{w}(v)\mathbf{w}(A(u,v))$ and

$$\begin{aligned}
\sigma(F) &= \frac{2}{k(k-1)} \sum_{u,v\in\mathcal{L}} \sigma(u,v) \\
&= \frac{2}{k(k-1)} \sum_{u,v\in\mathcal{L}} \sum_{x\in\mathcal{L}(A(u,v))} \mathbf{w}(u)\mathbf{w}(v)\mathbf{w}(x)\varepsilon(A(u,v),x) \\
&= \sum_{w\in T-\mathcal{L}} \sum_{u\in\mathcal{L}(\lambda(w))} \sum_{v\in\mathcal{L}(\rho(w))} \sum_{x\in\mathcal{L}(w)} \frac{2\mathbf{w}(u)\mathbf{w}(v)\mathbf{w}(x)\varepsilon(w,x)}{k(k-1)} \qquad (1)
\end{aligned}$$

By linearity of expectation, we can find expected value of each term and then add these expectations together.

For $u \in \mathcal{L}(\lambda(w))$, $v \in \mathcal{L}(\rho(w))$, $x \in \mathcal{L}(w)$,

$$\mathbf{E}\left[\mathbf{w}(u)\mathbf{w}(v)\mathbf{w}(x)\right] = \frac{k(k-1)(k-2)}{n(n-1)(n-2)} + [\![x \in \{u,v\}]\!] \frac{k(k-1)(n-k)}{n(n-1)(n-2)}$$

Thus

$$\mathbf{E}\left[\frac{2\mathbf{w}(u)\mathbf{w}(v)\mathbf{w}(x)\varepsilon(w,x)}{k(k-1)}\right] = \frac{2}{n(n-1)}\varepsilon(w,x)\left(\frac{k-2}{n-2} + [\![x \in \{u,v\}]\!]\frac{n-k}{n-2}\right)$$

The expected value of (1) is the summation of the above expressions that themselves are sums of two terms, $t_1(u,v,x) + t_2(u,v,x)$.

For every pair $\{u,v\} \subset \mathcal{L}$ we can add $t_1(u,v,x)$ for all $x \in \mathcal{L}(w)$ where $w = A(u,v)$; only $\varepsilon(w,x)$ is not the part of the common factor of these terms and, by our observation, the sum of $\varepsilon(w,x)$'s is 1, so the result is $2(k-1)/[n(n-1)(n-2)]$, when we add these results for all $n(n-1)/2$ such pairs we get $(k-2)/(n-2)$.

Now we add terms $t_2(u,v,x)$ that, for a fixed w, satisfy $A(u,v) = w$; note that in positive terms of that form $x \in \{u,v\}$:

$$\begin{aligned}
&\frac{2(n-k)}{n(n-1)(n-2)} \sum_{u \in \mathcal{L}(\lambda(w))} \sum_{v \in \mathcal{L}(\rho(w))} (\varepsilon(w,u) + \varepsilon(w,v)) \\
&= \frac{2(n-k)}{n(n-1)(n-2)} \sum_{u \in \mathcal{L}(\lambda(w))} |\mathcal{L}(\rho(w))|\varepsilon(w,u) + \sum_{v \in \mathcal{L}(\rho(w))} |\mathcal{L}(\lambda(w))|\varepsilon(w,v) \\
&= \frac{2(n-k)}{n(n-1)(n-2)} \sum_{u \in \mathcal{L}(\lambda(w))} |\mathcal{L}(\rho(w))|\frac{1}{2}\varepsilon(\lambda(w),u) + \sum_{v \in \mathcal{L}(\rho(w))} |\mathcal{L}(\lambda(w))|\frac{1}{2}\varepsilon(\rho(w),v) \\
&= \frac{(n-k)}{n(n-1)(n-2)}(|\mathcal{L}(\lambda(w))| + |\mathcal{L}(\rho(w))|) = \frac{(n-k)}{n(n-1)(n-2)}|\mathcal{L}(w)| \qquad (2)
\end{aligned}$$

One can see that $\sum_{w \in T-\mathcal{L}} |\mathcal{L}(w)|$ is equal to the sum of distances of leaves from the root of T, so if $\alpha(T)$ is the average distance of leaves of T to the root then the sum of (2) over all internal nodes of T is equal to $(n-k)/[(n-1)(n-2)]\alpha(T)$.

Summarizing, if F is a random set of leaves with k elements,

$$S(T,k) = \mathbf{E}\left[\sigma(F)\right] = \frac{k-2}{n-2} + \frac{n-k}{(n-1)(n-2)}\alpha(T)$$

where $n = |\mathcal{L}(T)|$ and $\alpha(T)$ is the average distance of leaves from the root.
